# Supplementary material for: Attosecond electron bunches from a nanofiber driven by Laguerre-Gaussian laser pulses
Source: Sci Rep. 2018 May 8;8:7282. doi: 10.1038/s41598-018-25421-9 (PMC5940694; doi:10.1038/s41598-018-25421-9)
Supplement: Supplementary file 1 — Supplementary information [file 41598_2018_25421_MOESM1_ESM.pdf]

Supplementary information: Attosecond electron bunches from a nanofiber driven by Laguerre-Gaussian laser pulses

Li-Xiang Hu[1], Tong-Pu Yu[1,2,*], Zheng-Ming Sheng[2,3,4], Jorge Vieira[5], De-Bin Zou[1], Yan Yin[1,6], Paul McKenna[2], and Fu-Qiu Shao[1]

[1]Department of Physics, National University of Defense Technology, Changsha, 410073, China
[2]SUPA Department of Physics, University of Strathclyde, Glasgow G4 0NG, UK
[3]Collaborative Innovation Center of IFSA (CICIFSA), Key Laboratory for Laser Plasmas (MoE) and School of Physics and Astronomy, Shanghai Jiao Tong University, Shanghai, 200240, China
[4]Tsung-Dao Lee Institute, Shanghai, 200240, China
[5]GoLP/Instituto de Plasmas e Fusão Nuclear, Instituto Superior Técnico, Universidade de Lisboa, 1049-001 Lisbon, Portugal
[6]Institute of Applied Physics and Computational Mathematics, Beijing, 100094, China
[*]Corresponding author: tongpu@nudt.edu.cn

ABSTRACT

This document provides supplementary information to "Stable attosecond electron bunches from a nanofiber driven by Laguerre-Gaussian lasers". It contains expanded simulation results and more discussions. It is shown that the electron energy peak is still clear at 330 fs. The robustness of the scheme has been also confirmed by the simulations with consideration of the front end pre-expansion. Meanwhile, the same physics occurs for a target with a rotation degree up to 11° with respect to the laser axis, which is more than sufficient accuracy for alignment in an experiment.

More simulation details

Figure S1(a) shows trajectories of some typical electrons until $t = 10T_0$. We see the return current continually pumps electrons to the left tip, and some of them close to the wire surface are dragged out by the radial electric fields. Figure S1(b) exhibits the energy spectrum of the attosecond electron bunch at $t = 100T_0$. The energy peak at 320 MeV is still clear after such a long time propagation. Figure S1(c) presents the total number and average energy of electrons dragged out from different nanowire locations. About 61.3% of the attosecond electrons originate from the wire position of $3 \leq x/\lambda_0 \leq 4$, and their average energy is up to 120 MeV at $t = 35T_0$. Figure S2 presents the electron distribution in the (y, θ_y) and (z, θ_z) space at $t = 60T_0$. The corresponding transverse beam root-mean-square emittance ε_y and ε_z are 1.4×10^{-2} mm·mrad and 1.5×10^{-2} mm·mrad, respectively.

Effect of the pre-pulse and laser pointing

Figure S2 presents the simulation results with consideration of the front end pre-expansion. In this simulation, we assume a cone-like pre-expansion of wire with the density linearly increasing from $5n_c$ to $20n_c$ over a distance of $5\lambda_0$. As shown in the figure, the central electron bunch is dense and intact, with maximal density up to $6n_c$ at $t = 30T_0$. As compared to the Fig. 1(e) without consideration of the wire expansion in the primary manuscript, the electron density is at the same level.

We also consider the effect of laser pointing, e.g., the nanowire is rotated by a small angle with respect to the laser axis, as shown in Fig. S3. In order to keep the electrons in phase of the laser fields, the wire electrons shoud be located within the region of $r < r_m$, where r_m represents the position of the maximum radial electric field E_r. Thus the angle should be smaller than the value $\arctan[(r_m - R_0)/(0.5L_0)]$. Taking the parameters in our manuscript, we find that the same physics occurs for a target with a rotation degree up to 11°, which is more than sufficient accuracy for alignment in an experiment.

An example simulation with a much lower laser intensity

Figure S4 presents the details of the case with $a_0 = 10$, which corresponds to a laser intensity of 10^{20} W·cm^{-2}. We see that an attosecond electron bunch train is also generated in this situation, with the maximum electron energy up to 180 MeV. The total

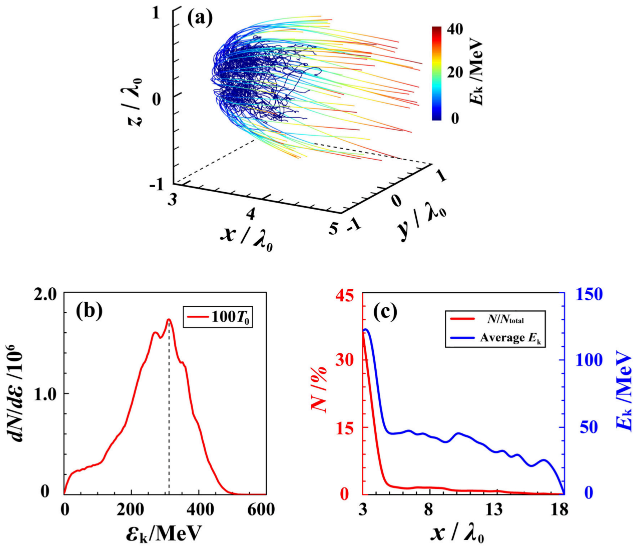

Figure S1. (a) Trajectories of typical electrons until $t = 10T_0$. (b) Energy spectrum of the attosecond electron bunch as marked in Fig. 1 in the manuscript at $t = 100T_0$. (c) Distributions of electron number and average energy versus their initial positions along the nanowire at $t = 35T_0$.

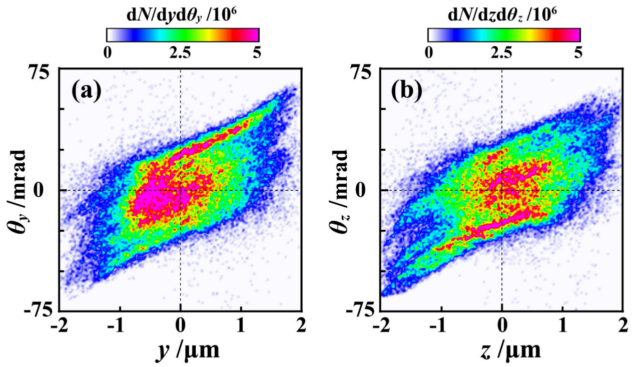

Figure S2. Electron distribution in the (a) (y, θ_y) and (b) (z, θ_z) space at $t = 60T_0$.

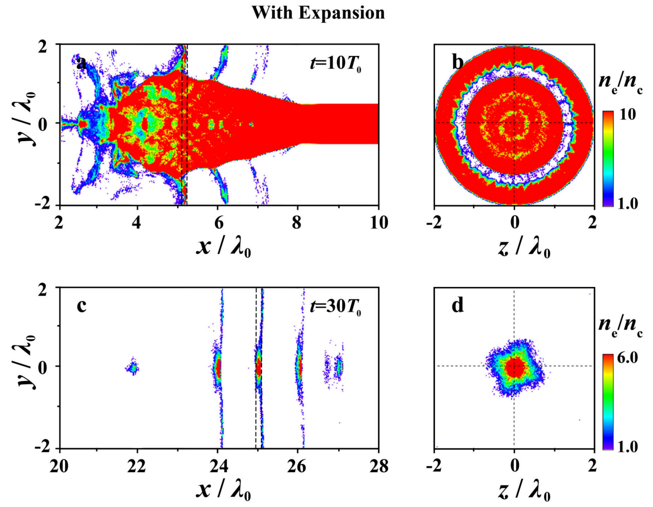

Figure S3. Testing the effect of the pre-pulse. The electron density distribution in the xy (a, c) and yz (b,d) plane when taking into account the target expansion due to prepulse laser interaction at $t = 10T_0$ and $30T_0$, respectively.

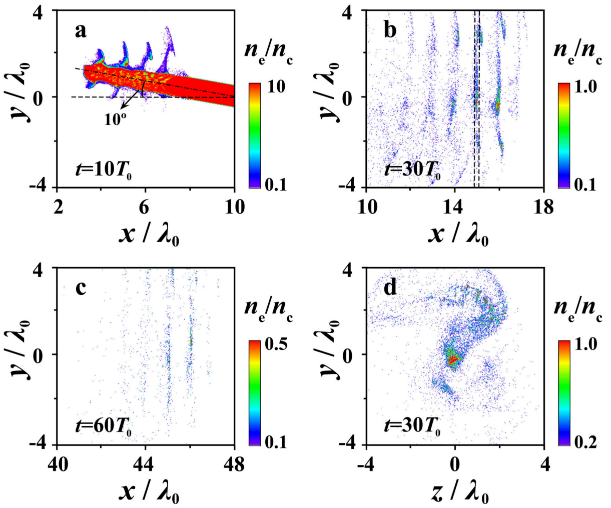

Figure S4. The electron density distribution in the xy (a, b, c) and yz (d) plane at $t = 10T_0$, $30T_0$ and $60T_0$ when taking into account the effect of the laser pointing.

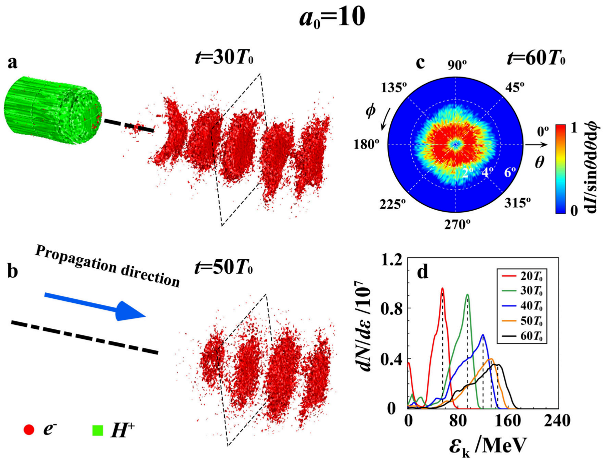

Figure S5. 3D PIC simulation results for the case with $a_0 = 10$. (a,b) The electron density evolution. (c) The divergence angle distribution (at $t = 60T_0$) and (d) the energy spectrum of the bunch as marked in (a,b).

beam charge is 0.3 nC, which is still much larger than in the normal laser wakefield bubble regime[1].

References

1. Pukhov, A. & Meyer-ter-Vehn, J. Laser wake field acceleration: the highly non-linear broken-wave regime. *Appl. Phys. B* **74**, 355-361 (2002).
